# Chordoma dedifferentiation after proton beam therapy: a case report and review of the literature

**DOI:** 10.1186/s13256-016-1076-3

**Published:** 2016-10-12

**Authors:** Joseph Frankl, Cassi Grotepas, Baldassare Stea, G. Michael Lemole, Alexander Chiu, Rihan Khan

**Affiliations:** 1 University of Arizona College of Medicine, 1501 N Campbell Ave, Tucson, AZ 85724 USA; 2 Department of Pathology, University of Arizona College of Medicine, 1501 N Campbell Ave, Tucson, AZ 85724 USA; 3 Department of Radiation Oncology, University of Arizona College of Medicine, 1501 N Campbell Ave, Tucson, AZ 85724 USA; 4 Department of Surgery, University of Arizona College of Medicine, 1501 N Campbell Ave, Tucson, AZ 85724 USA; 5 Department of Otolaryngology – Head and Neck Surgery, University of Arizona College of Medicine, 1501 N Campbell Ave, Tucson, AZ 85724 USA; 6 Department of Medical Imaging, University of Arizona College of Medicine, 1501 N Campbell Ave, Tucson, AZ 85724 USA

**Keywords:** Chordoma, Proton beam therapy, Dedifferentiation, Pediatric tumors, Oncology, Case report

## Abstract

**Background:**

Chordoma is a rare invasive bone tumor that may occur anywhere along the neuraxis. A total of three primary histological varieties have been identified: conventional, chondroid, and dedifferentiated.

**Case presentation:**

We report a case of an 8-year-old white girl who presented with conventional chordoma, was treated with surgical resection and mixed proton and photon beam therapy, and had a recurrence in the resection cavity 2.5 years later with dedifferentiated morphology. The recurrent tumor did not express brachyury, a recently identified protein specific to tissue of notochordal origin.

**Conclusions:**

The short time period between radiation therapy and dedifferentiation, low dose of photons, and rarity of dedifferentiated skull base chordomas in pediatric patients should alert clinicians to the possibility of chordoma dedifferentiation after proton beam therapy.

## Background

Chordoma is a rare, locally invasive bone tumor of notochordal origin with an incidence of 0.1 to 0.8/1,000,000 [1–3]. It is 40 % less likely in females compared to males [2, 4]. The tumor can present at the base of the skull, in the vertebral column, or in the sacral region, but there is controversy over the relative incidence at these sites [2, 5]. Although chordomas are typically a tumor of the aged, presentation in younger individuals tends to occur at the skull base [2, 4, 6]. In addition, female gender is associated with greater likelihood of presentation in the skull base [2, 4]. The overall median survival after chordoma diagnosis is approximately 6 years, but it is higher for tumors with chondroid histology and lower for those with dedifferentiated histology compared to the conventional variety [2, 7].

Skull base chordomas are typically surgically resected and proton beam therapy is often used to treat residual tumor [8, 9]. We report the first case, to the best of our knowledge, of a skull base chordoma that dedifferentiated after proton beam therapy. A timeline of the case is shown in Table 1.

**Table 1:** Timeline of the patient’s clinical course

Date	Event
16 Jun 2012	Initial presentation with headaches and emesis prompts magnetic resonance imaging that shows a large heterogeneous skull base mass suspicious for chordoma.
2 Jul 2012	Endonasal surgical resection. Surgical pathology confirms chordoma diagnosis.
2 Nov – 29 Nov 2012	Fractionated proton and photon beam therapy: 77.4 Gy total, 59.4 Gy (cobalt gray equivalent) in 33 fractions with protons and 18 Gy in 10 fractions with photons.
18 Jun – 11 Dec 2013	Interval decrease in tumor size visualized at scheduled magnetic resonance imagings.
3 Jan 2015	Visual disturbances prompt magnetic resonance imaging, which shows an enhancing mass in the resection site suspicious for recurrence with dedifferentiation.
21 Jan 2015	Retrosigmoid craniotomy. Surgical pathology shows complete dedifferentiation.
29 Mar 2015	Begins ifosfamide and etoposide alternating every 2 weeks with vincristine, doxorubicin, and cyclophosphamide.
23 Apr 2015 – 25 Mar 2016	Interval decrease in tumor size visualized at scheduled magnetic resonance imagings. Continues ifosfamide and etoposide alternating every 2 weeks with vincristine, doxorubicin, and cyclophosphamide.
26 Apr 2016	Last of 17 cycles of ifosfamide and etoposide alternating with vincristine, doxorubicin, and cyclophosphamide. Begins maintenance capecitabine.

## Case presentation

### Clinical course

An 8-year-old white girl with no family history of skin cancer except for skin cancer in her grandfather complained of 4 weeks of pressure-like headaches, mostly in the posterior occipital area, and intermittent nausea with several episodes of emesis in June of 2012. A physical examination was significant for decreased sensation to light touch over the right side of her face and her right upper extremity. Magnetic resonance imaging (MRI) of her brain revealed a large heterogeneous skull base mass. Chordoma was suspected over chondrosarcoma, and the diagnosis was confirmed by pathology after tissue was obtained during an endonasal surgical resection. Resection of the chordoma left residual tumor in her lateral skull base at the hypoglossal canal and jugular foramen. She was referred to a proton facility where she received fractionated proton and photon beam therapy: 77.4 Gy total, 59.4 Gy (cobalt gray equivalent) in 33 fractions with protons and 18 Gy in 10 fractions with photons. She was returned to our care afterwards. Scheduled MRIs showed decreasing lesion size through postoperative year 2. However, she experienced acute cranial nerve 6 palsy approximately 2.5 years after surgical resection. An MRI showed a 3×1.5×4 cm mass in the resection cavity with signal change suspicious for dedifferentiation. A subsequent biopsy obtained during a retrosigmoid craniotomy confirmed high-grade sarcoma. She has since completed 17 cycles of ifosfamide and etoposide alternating with vincristine, doxorubicin, and cyclophosphamide with the last round in April 2016. She began maintenance capecitabine on 26 April 2016. The most recent MRI, approximately 14 months after her second surgery, showed a mild interval decrease in tumor size.

### Imaging findings

**Fig. 1:**
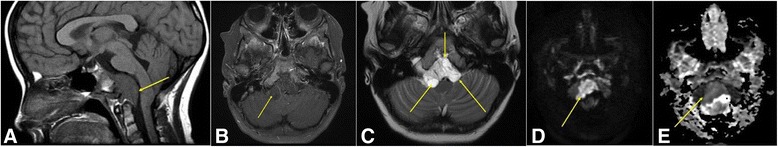
**a** T1-weighted sagittal magnetic resonance imaging showing a lesion isointense to muscle which displaced pons and medulla posteriorly and with a small nasopharyngeal component (*arrow*). **b** Post-contrast axial T1-weighted image showed mild enhancement (*arrow*). **c** Axial T2-weighted magnetic resonance imaging showing bright signal (*arrows*). **d** Restricted diffusion with bright diffusion-weighted imaging signal (*arrow*). **e** Restricted diffusion with dark apparent diffusion coefficient signal (*arrow*)

MRI of our patient’s brain with and without contrast at her initial presentation showed a large heterogeneous mass emanating intracranially from the base of her clivus and extending through the foramen magnum to C1–C2. A small portion was anterior to C1–C2 in her nasopharyngeal soft tissues with extension through her left hypoglossal canal. Posteriorly, the lesion displaced her pons and medulla. Multiple cranial nerves were not well seen and were either displaced or encased by the mass as it filled the right cerebellomedullary angle. The lesion was T1 isointense to muscle (Fig. 1a), and only mildly enhanced in portions (Fig. 1b). The lesion had a predominantly very bright T2 signal (Fig. 1c). There was restricted diffusion with bright diffusion-weighted imaging and dark apparent diffusion coefficient signals, indicating hypercellularity (Fig. 1d, e).

**Fig. 2:**
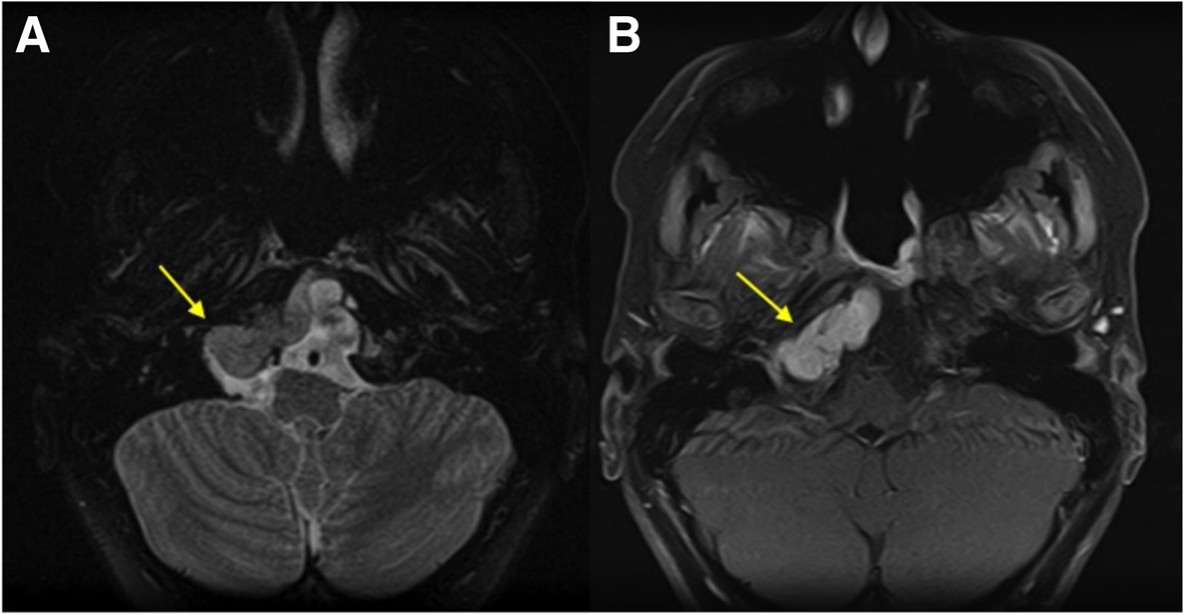
**a** Axial T2-weighted image of the recurrent tumor showing intermediate to dark, isointense to gray matter signal intensity (*arrow*). **b** Axial T1-weighted post-contrast image showing avid enhancement (*arrow*)

Follow-up MRI 2.5 years after the initial MRI, and after surgery and proton beam therapy, showed local recurrence. The recurrent tumor was centered at the rightward aspect of her clivus and extended superiorly to the dorsum sella and inferiorly to the level of the occipital condyle. The signal intensity of this mass was intermediate to dark, isointense to gray matter on T2-weighted images in contrast to the very T2 hyperintense signal like cerebral spinal fluid as seen on the initial presentation (Fig. 2a). In addition, on follow-up the lesion was avidly enhancing post-contrast on T1-weighted imaging (Fig. 2b) as compared to the initial lesion where there was only mild enhancement.

### Pathology findings

**Fig. 3:**
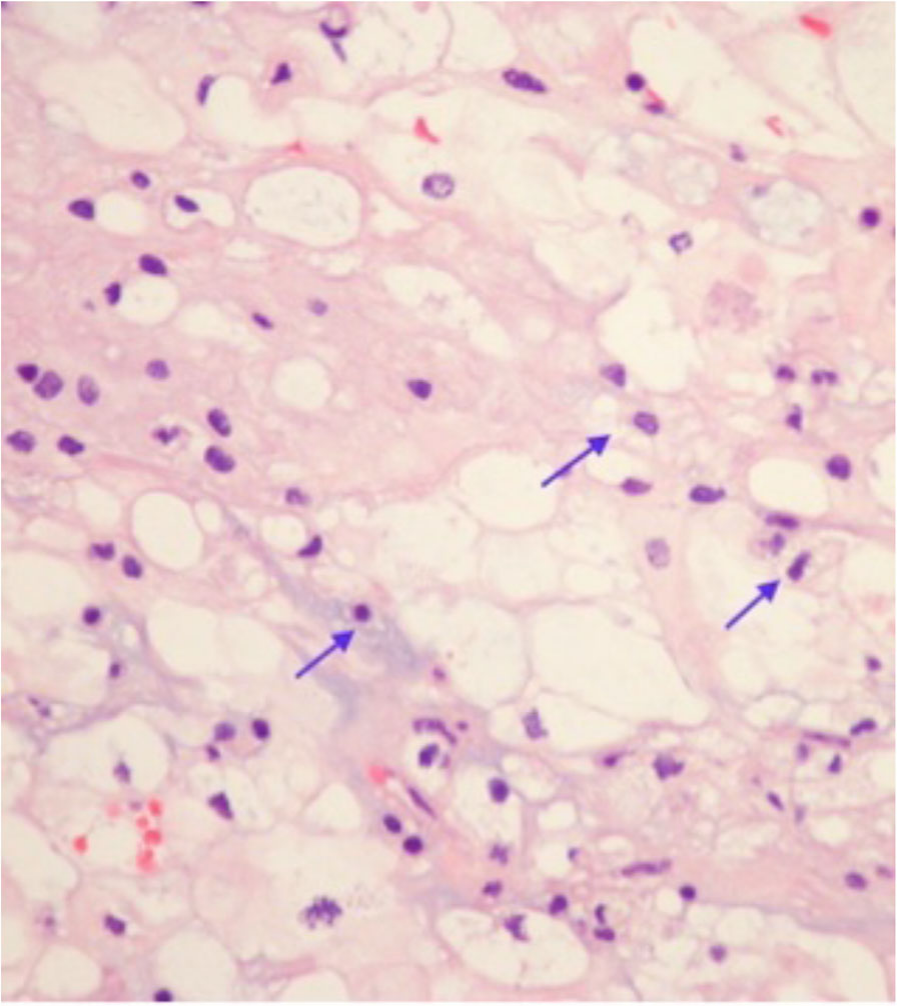
Hematoxylin and eosin stain showing pathognomonic physaliphorous cells with multivacuolated cytoplasm (*arrows*), mixed with epithelioid cells. Nuclei are relatively bland without significant pleomorphism. Magnification 40×

The biopsy from the initial lesion showed classic chordoma histology with physaliphorous cells with multivacuolated cytoplasm mixed with epithelioid cells arranged in anastomosing cords, clusters, and chains. There was a myxoid matrix and occasional fibrous septa (Fig. 3). No immunohistochemical stains were performed as histology provided a definitive diagnosis.

**Fig. 4:**
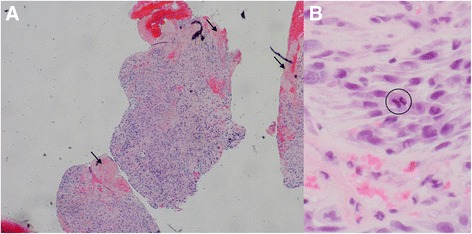
**a** Unlike tissue from the first biopsy, the recurrent tumor had prominent areas of necrosis (*arrows*; 5× magnification). **b** Mitotic figure (*circled*) and nuclear pleomorphism were seen at 40× magnification. Spindle cells were seen, but physaliphorous cells were not noted

Hematoxylin and eosin staining of the recurrent tumor obtained during our patient’s second surgery, however, showed a high-grade anaplastic spindle cell neoplasm. There were areas of geographical necrosis with no particular architectural pattern visible at low magnification (Fig. 4a). In the higher power images, the cells were very pleomorphic with epithelioid and spindle shapes, and there were mitotic figures present (Fig. 4b). Myofibroblastic tumor and fibrosarcoma, leiomyosarcoma, and rhabdomyosarcoma were ruled out with immunohistochemistry. Vimentin was positive, consistent with sarcoma in general. Ki-67, a proliferation index that is normally very low in a chordoma, was scored at 80 %, indicating rapid cellular division. Epithelial membrane antigen (EMA), cytokeratin, and brachyury were negative, indicating full dedifferentiation.

## Discussion

Less than 5 % of chordoma diagnoses occur in children under 10 years of age [2]. Among childhood cases, Borba and colleagues found a distinct difference in presentation, tumor morphology, and prognosis between cases occurring in children under and over age 5 [10]. Children under age 5 frequently have long tract deficits [11, 12] and intracranial hypertension, and the tumor is often of an atypical morphology, which has a poor prognosis [10]. Children over age 5 most frequently present with double vision [13–17] and headaches [13, 18, 19], have typical tumor morphology, and, thus, survive for longer [10]. Our patient, an 8-year-old girl presenting with headaches and nausea, was found to have a typical clival chordoma, which initially fit the expected pattern of disease presentation. The histology and imaging appearance of her tumor, though, changed with recurrence 2.5 years after the initial surgical resection and radiation therapy.

Chordomas show dual epithelial-mesenchymal differentiation as they arise from embryonic remnants of the notochord [20]. Although the molecular pathogenesis of chordoma is not fully known, brachyury has been shown to be both a regulator of notochordal differentiation and a chordoma marker. Cytogenetic studies have shown that mutations on multiple chromosomes are associated with chordoma [21]. Conventional chordomas feature large polygonal cells with abundant eosinophilic to amphophilic cytoplasm, variable nuclei, and occasional vacuoles (physaliferous cells) arranged in cords and sheets in a myxoid stroma [22, 23]. Chondroid chordomas have regions where the stroma resembles hyaline cartilage and neoplastic, sometimes physaliferous, cells grow in lacunae [22, 23]. Finally, dedifferentiated chordomas have regions of or have become entirely composed of malignant sarcomatous cells [22, 24]. Previous reports have shown possible loss of EMA and cytokeratin [24, 25], but our case is the first to report absence of brachyury in the dedifferentiated tumor. In addition, dedifferentiation was faster than in earlier reports of completely dedifferentiated chordoma occurring after conventional radiotherapy [26, 27].

T1-weighted MRI shows chordomas as having predominantly intermediate to dark signal [28]. They are typically bright on T2-weighted MRI, but dedifferentiated chordomas are not [29]. Diffusion-weighted MRI will show diffusion restriction, but this may be difficult to interpret in skull base tumors due to artifact at the skull base [30]. Contrast enhancement is variable [31]. Further advancements in molecular imaging may allow for definitive diagnosis without the need for biopsy. Much as immunohistochemistry is used to identify proteins in biopsies, radiolabeled molecular probes and targeted MRI contrasts may be used in the future to identify unique proteins in neoplasms [32]. Skull base chordomas most often involve the upper half of the clivus and often extend to the lower half of the clivus, posterior clinoid process, cavernous sinus, and occipital condyle [30]. Our patient’s tumor showed typical MRI presentation when she first presented to us. However, the resection site recurrence had intermediate to dark signal on T2-weighted images.

Most recent research indicates the overall prognosis for younger patients is better than for adult patients with chordoma, although there have been contradictory findings [3, 4, 23, 30]. Surgical margins are the most important prognostic indicator for patients without dedifferentiated chordoma, and gross resection or debulking is recommended for patients without contraindications [8]. However, dedifferentiated chordomas are less responsive to surgery and carry a worse prognosis [8, 33]. Residual tumor in the resection cavity responds best to high doses of radiation, which creates a difficulty when treating clival tumors because the adjacent structures are critical and radiosensitive [8]. Proton beam therapy is used to deliver high doses of radiation (70 to 80 GyE) with less damage to non-neoplastic tissue than conventional photon radiotherapy due to its sharp Bragg peak [9, 34]. However, side effects such as radiation necrosis of the optic nerve and temporal lobe, epilepsy, inhibition of the hypothalamic–pituitary axis, and other endocrinopathies have been reported [8, 34, 35]. Hara and colleagues reported the only case of a sarcomatoid transformation after proton beam therapy, which occurred 3 years after the initial treatment and 1 year after a second course of proton beam therapy [36]. It is important to note that some authors distinguish between sarcomatoid chordoma and dedifferentiated chordoma in that the first retains immunoreactivity for cytokeratin, whereas the latter does not [24, 37].

## Conclusions

Our patient’s case is the first report, to the best of our knowledge, of a chordoma that dedifferentiated after protein beam therapy. There are reports of dedifferentiation after conventional radiation therapy, although the typical photon dose in those cases is severalfold greater than 18 Gy and disease progression much longer than 2.5 years [27, 38, 39]. The case reported here should alert clinicians to the possibility of dedifferentiation after proton beam therapy and prompt histologic analysis of recurrent tumors in this setting. We did not identify any other cases of dedifferentiated skull base chordoma in pediatric patients when reviewing 29 cases published after Borba and colleagues’ 1996 literature review [10]. This highlights the unusual nature of this presentation and the likelihood that proton beam therapy may have contributed to the tumor’s dedifferentiation.

## Abbreviations

EMA: Epithelial membrane antigen
MRI: Magnetic resonance imaging
